# Comparative Study of Selected Order-Picking Methods: Efficiency, Ergonomics, and Adaptation Rate of New Employees

**DOI:** 10.3390/s25030923

**Published:** 2025-02-03

**Authors:** Marcin Łopuszyński, Kamil Janusz, Dawid Karwat

**Affiliations:** Faculty of Information and Communication Technology, Wroclaw University of Science and Technology, ul. Janiszewskiego 11/17, 50-372 Wrocław, Poland

**Keywords:** order picking, pick-by-light, pick-by-paper, pick-by-point, efficiency

## Abstract

The human manual order-picking process in the warehouse is still the leading method despite increasing automation. This manual process is supported by indicating and receipt systems to reduce the order-picking time and the number of errors. Many studies in the literature compare the Pick-by-Light system with the Pick-by-Paper and other systems, and it is more challenging to study the Pick-by-Point system. This paper presents the results of laboratory comparative studies of the most straightforward Pick-by-Paper system with Pick-by-Light and Pick-by-Point systems supported by receipt systems. In the case of the Pick-by-Light system, the receipt system is a button on the module that the picker presses to confirm the pick-up of an item. In the case of Pick-by-Point, the receipt system is a wrist scanner that the picker uses to confirm the pick-up of an item. A total of 71 people participated in the study. Participants completed five orders with five items per system. Comparisons were made of the time it took to pick the orders with the support of these systems, the number of errors made, ergonomics, and the speed of adaptation for new employees without experience. A person with nine years of experience in the picking process took part in the study, whose order-picking times were compared with those of the others. In the study, the Pick-by-Light system proved to be the fastest regarding order picking and the adaptation of new employees. On the other hand, the Pick-by-Point system was the most error-proof.

## 1. Introduction

The picking process is an essential part of the more extensive process, which is the logistics of transporting goods from the warehouse to the customer or parts and raw materials from the warehouse to the production line. The efficiency of this system is affected by factors such as the arrangement of items in the warehouse space and the path of pass of the warehouse worker, among others. Methods for optimizing these factors are described by M. Garbacz et al. [1,2]. An extensive survey of publications from several angles and, in particular, methods for determining the paths of a worker’s pass to pick an order was conducted by A. Setayesh et al. [3]. Another broad review of publications issued between 2007 and 2022 in the field of order-picking systems, including 269 publications, was compiled by G. Casella et al. [4] and Ch. Glock et al. [5]. The development of robotics and control has had a significant impact on the introduction of systems without human involvement to eliminate errors that he may make [6]. However, man still plays a vital role in picking, assisted by information systems.

The first system that supported humans in the picking process was using a piece of paper. Such a document has a list of items, their quantity, and sometimes even the location of where these items are. Today, this method is often called Pick-by-Paper. The correctness of picking by this method depended on the accuracy of the worker as well as the orderliness of the warehouse and the preparation of the paper document. To eliminate errors and facilitate employees’ work, light effects were introduced to guide them to the correct location. Modules with lights also began to be equipped with buttons to confirm that an article had been picked from the indicated position. This system is now called Pick-by-Light.

Several publications can be found in the literature, in which the results of comparative studies of the systems mentioned above are presented. For example, Ch. Stockinger et al. [7] presented the results of a comparative study of Pick-by-Light and Pick-by-Paper systems conducted on a sample of 31 people. The average age of the subjects was 30. The study tested the participants’ situational awareness and blood pressure after the order was collected. In contrast, in a publication by A. Baechler et al. [8], the authors tested Pick-by-Paper, Pick-by-Light, Pick-by-Display, and Pick-by-Projection systems. One of the goals of this study was to test the speed of the picking process using the systems above. Twenty-four people (sixteen men and eight women) between the ages of 20 and 54 participated in the study. Among the participants, four had a technical or academic background. The system that allowed the fastest order picking was the Pick-by-Light system. The errors made were also studied. There was no significant difference here, but the fewest errors were made with the Pick-by-Light and Pick-by-Projection systems. The Pick-by-Paper and Pick-by-Light methods were also studied by G. Anhong et al. [9]. The authors studied order picking using four methods: Pick-by-Paper, Pick-by-Light, Pick-by-CMD, and Pick-by-HUD. The first method is traditional picking with a piece of paper, with the locations of the items to be picked and the quantities specified on it. In the second method, luminous modules indicate where the article should be picked up from. With Pick-by-CMD (Cart-Mounted Display), the picking list is presented graphically on a display mounted on the picking cart. With Pick-by-HUD (Head-Up Display or Head-Mounted Display), the graphical picking list is presented on a display mounted on the participant’s head. The publication’s authors do not mention the receipt of the article to be taken. Eight students participated in the study. The study was divided into a training part and an actual part. The results showed that the slowest method was the traditional method using paper. It was followed by the Pick-by-Light method. The authors also studied the evaluation of the comfort and ease of learning the method, as well as the number of errors made. The Pick-by-Paper method fared worst in each criterion. In contrast, the ease of learning for the Pick-by-Light method was rated at the same level as the Pick-by-HUD and Pick-by-CMD methods. For comfort of use, the Pick-by-Light method was rated second along with the Pick-by-HUD method. According to participants, Pick-by-CMD was the most comfortable method. The Pick-by-Light method, however, fared the worst in terms of errors made. The traditional paper method proved to be more accurate. An example of a comparative study of the traditional paper-based system with the Pick-by-Light system but in a manufacturing process was presented by J. Trojanowska et al. [10]. The publication presented the results of a study of a specific extruder assembly process—the Pick-by-Light system aimed to reduce part-picking errors and shorten the process time. A 7% to 35% reduction in process time for individual workers and a 35% decrease in picking errors were achieved.

The use of augmented reality glasses has also been tried in the picking process. A comparative study of systems such as Pick-by-HUD, which involves using Google Glass glasses to display information on their lenses, with the Pick-by-Light system was conducted by Wu Xiaolong et al. [11]. In terms of speed, the Pick-by-HUD system proved superior. In contrast, the study showed less susceptibility to errors made with the Pick-by-Light method. The methods studied did not have a receipt system. Other results of a survey of a system using glasses conducted by K.A. Weaver were obtained [12]. This study compared a system using Sony glasses with the Pick-by-Paper method in two variants (text and graphic) and the Pick-by-Voice system, which involves voice messaging to the employee. Twelve people participated in the study. This study indicates that the picking time was shorter with the use of glasses, while the voice system proved to be the slowest.

The above studies did not use receipt systems. A study that uses a receipt with a scanner placed on a glove was carried out by C.S. Murauer as part of her doctoral thesis in [13]. However, the author did not compare the receipt system with any other. Instead, she used different variants of the information displayed in augmented reality glasses for indicating. A comparison of receipt systems without indicating systems can be found in the work of C. Scheuermann et al. [14]. The authors compared a traditional handheld scanner with a glove-mounted wrist scanner in two variants: on one hand and two hands. The results of these studies indicate a shorter picking time with the wrist scanner. P. Fager also compared the receipt systems themselves and the results published in [15]. The publication compared a push-button receipt system on a Pick-by-Light module attached to a rack shelf, a bracelet with an RFID wrist reader, a barcode wrist scanner, and a voice. Four people with no picking experience participated in this study. As a result of this study, the fastest system proved to be the button receipt on the module. There were 167 incorrect pick-ups recorded out of a total of 1 million. A study of picking using indicating and receipt systems was carried out by Ch. Thomas et al. in the publication [16]. The systems compared were Pick-by-Paper with a receipt by hand scanner, Pick-by-Light with a push button, Pick-by Paper without a receipt, and Pick-by-HUD with an RFID scanner. In addition to studying the duration of the process, the authors also investigated the number of errors made. In the study, each 12 participants completed 10 tasks with each method. Pick-by-Paper with a scanner proved to be the slowest system. The Pick-by-Light and Pick-by-HUD systems had very similar times. Regarding errors, the Pick-by-Light and Pick-by-HUD systems yielded 30 incorrect pick-ups each. Pick-by-Paper with scanner yielded 59. In contrast, picking with Pick-by-Paper without receipt yielded 161 errors.

Among the available studies, it is not easy to find studies that use a Pick-by-Point or similar system, i.e., a system that uses a single lamp per zone in the warehouse and indicates with a light beam where the item will be picked up. A publication presenting pilot studies using a typical Pick-by-Point system is that of A. Wrobel et al. in [17]. Eight systems were tested in the study, the fastest of which were two Pick-by-Point variants with a scanner and a hand-held push-button remote control. Similar average times were obtained for the two variants of the Pick-by-Light system, i.e., with a scanner and a button on the module. Some similarities to the Pick-by-Point system could be seen in the Pick-by-Projection system described by B. Baechler et al. in [8]. A Pick-by-Projection system, which is somewhat similar to Pick-by-Point, was also tested by M. Funk et al. in [18]. Pick-by-Paper, Pick-by-Voice, and Pick-by-Vision systems were also tested. These tests showed that the Pick-by-Projection system was more resistant to errors made than the others and gave picking times similar to those of the Pick-by-Voice system. In contrast to the system presented in [8,18], Pick-by-Point is more straightforward. It uses only the lamp. Similarly, it is difficult to identify more studies comparing complete picking systems, i.e., indicating and receipt systems. This type of study is conducted internally within manufacturing companies and is not published externally.

The publications mentioned here present studies of indicating systems and receipt systems. Often, the studies are one of two. The studies mainly boil down to an analysis of the duration of the process and the number of errors made by the participants. The speed of adaptation of new employees to a particular system and its ergonomics is also an essential aspect of the picking process. This is also the purpose of the study, the results of which are presented in this article. Even though studies on the effectiveness of systems supporting the manual picking process have been carried out for many years, the publication by J. Trojanowska et al. [10] shows the continuing relevance of this issue and the wide possibilities of application.

The novelty of this study is comparing the Pick-by-Point system with the Pick-by-Paper and Pick-by-Light systems in terms of picking speed and number of errors made. In addition, the adaptation of new employees to these systems is studied and compared. Another novelty is an ergonomic study based on the participants’ opinion of the Pick-by-Point system. Complete systems are studied, i.e., indicating (Pick-by-Point) and receipt (by a wrist scanner).

## 2. Method

### 2.1. Objectives

The purpose of the study is to compare selected systems used in the picking process specifically in three aspects:

- Comparison of systems in terms of speed of order picking and errors made by employees. The speed of order picking is the leading indicator examined when comparing systems. Similarly, the number of errors made, i.e., picking the wrong items or items from the wrong location.
- The speed of adaptation of people without experience to the selected systems. This is very important in the e-commerce industry because the most significant quantities of goods flow out of warehouses on the occasion of Santa Claus or Christmas, for example, when customers shop for gifts. There are periods in warehouses when there is a much lower load, and then there is no need to keep excess employees involved in the picking process. During peak periods, new employees who do not have much time to gain experience are often hired, so systems are sought that will be easily assimilated by inexperienced workers and will reduce errors made.
- Evaluation of the ergonomics of the tested systems by survey participants. This indicator is essential in addition to the above because of the impact on employee well-being, which can further affect time and erroneous downloads.

### 2.2. Used Concepts

In the article, the authors use terms with the following meanings:

- System—a set of tools to support picking (collecting orders in a warehouse).
  - Pick-by-Paper—picking an order with the help of a paper document.
  - Pick-by-Light—order picking supported by light modules placed on racks, receipt by button on module. The system works in step-by-step mode, i.e., only one location is indicated at a time.
  - Pick-by-Point—order picking supported by rotating light, receipt by wrist scanner.
- Pass—picking of an order. Pass means collecting one order of five items in a different location. There are five orders to complete within one system. The same orders in the same order are processed using three methods, i.e., three systems.
- Time—the time it takes to collect an order. In the study, this is the time from crossing the starting line to reaching the finish line.
- Error—retrieving an item from the wrong location.

### 2.3. Description of the Method

The study method used to conduct the study is as follows:

- Six months earlier, a pilot study was carried out with three people to eliminate errors in the study method. The result of this study was a reduction in the number of orders to be picked from 7 to 5. The main limitation was the time available for the study. A single group of students participated in the study during their class, which lasted 100 min.
- Initially, training was given to groups of 10–18 people on the warehouse layout, aisle direction, and shelf labeling. Each group was trained individually. The training took place before each system was tested.
- One system tested on one day. (In the case of part-time students, Pick-by-Paper was used on the first day, with Pick-by-Light and Pick-by-Point on the second day.) This was due to the smaller size of the groups, and therefore more time was available.
- Each person picked five orders one by one as part of the study of a particular system.
- Each order contained five items of one item each, unique to other orders. This means unique locations in each order. Each order had items from both the left and right racks. The list of orders and the order in which the locations were indicated was organized in a forward mode, meaning that the participant was moving forward all the time. There was no indicating backwards.
- The same orders were tested in the same order in each system.
- Participants were not allowed to peek at others during the picking of the orders and thus could not learn by looking.
- Incorrect pick-ups were counted. The sum of errors made by a given participant within a given system, i.e., all five passes, was counted. A member of the study staff counted the data.
- Four people were involved in handling the study. They did not participate in the study as participants.

Ergonomics survey

- Ergonomics surveys were carried out using short questionnaires.
- System-specific questionnaires were completed by participants immediately after completing the five orders to gather participant feedback.
- Comparison questionnaires were completed after the completion of all systems. The questionnaire was completed second after the questionnaire for the system currently under investigation.

### 2.4. Warehouse Infrastructure

**Figure 1:**
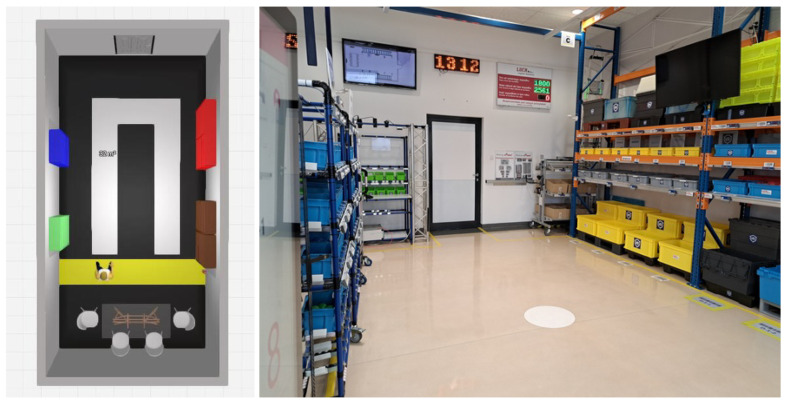
The testing laboratory layout and its photo.

**Figure 2:**
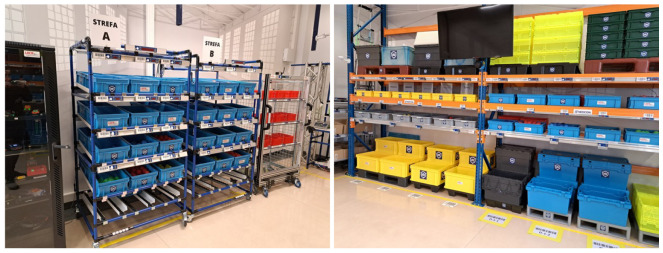
The shelves of the laboratory on the left (zones A and B) and the shelves on the right (zones C and D).

The testing place—a warehouse, where the tests were carried out, was the laboratory (Figure 1), which was equipped with four zones, with two on each side of the laboratory (Figure 1). Two zones, A and B, were on the left side of the laboratory, and two, C and D, were on the right (Figure 2). All zones were equipped with the devices used in the tested systems. The participant picked the order by walking clockwise first on the left side of the laboratory and returning on the right side.

Items and the cart

**Figure 3:**
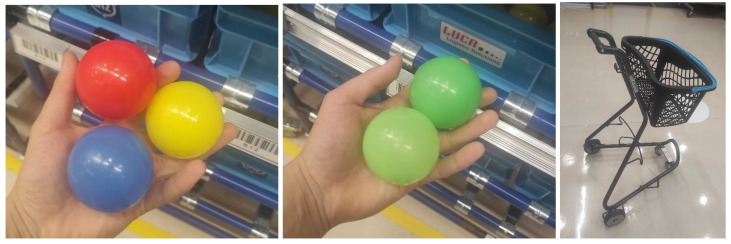
The balls that were in the bins and the cart to collect them.

Participants had to pick balls (Figure 3). Each item was one ball.

Only one ball had to be picked from each location and placed in the cart basket (Figure 3). The attendant emptied the cart basket after five orders had been completed by the participant and put back in the bins on the shelves.

### 2.5. Tested Systems

#### 2.5.1. Pick-by-Paper

**Figure 4:**
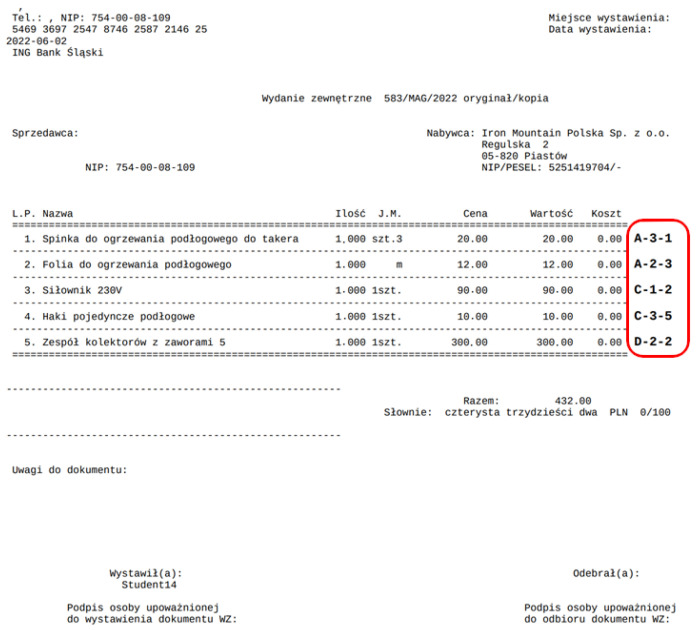
One of the five documents used in the Pick-by-Paper method.

The Pick-by-Paper order-picking method where balls were picked from the appropriate locations given in the document as in Figure 4. Each participant received the corresponding document before each pass. The orders were consistently completed in the same sequence.

**Figure 5:**
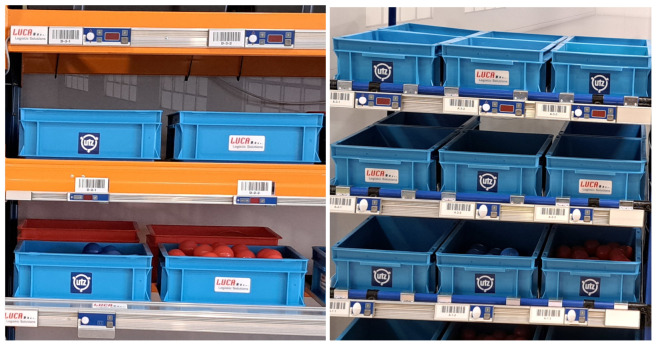
Pick-by-Light system modules with one or more buttons.

The addresses of the containers and bins were referred to as D-3-5. D stands for zone D. The number 3 is the shelf number counted from the bottom. This means that shelf 1 is the lowest. The number 5 is the bin number on shelf 3. The bin number is counted from the left to the right. The address is indicated on the label under the barcode next to the module of the Pick-by-Light system (Figure 5). In zones A and B, the label with the barcode and address is always above the bin. In zones C and D, the labels for containers on level 1 are on the floor (Figure 2). For the other bins, the labels are the same as in zones A and B, so above the bin.

#### 2.5.2. Pick-by-Light

In the Pick-by-Light system, the indicating devices are the lights on the modules (Figure 5), which in all zones are located above the container. The item pick receipt system is a button on the module next to the light. The participant in the study should mark the receipt of the pick-up after taking the item from the container and placing it in the cart basket. If the item pick-up receipt is marked with the button on the wrong module, the lamp on the module at the correct location starts flashing and turns red. This indication of incorrect pick-up is important if the receipt is carried out using, for example, an infrared curtain. If the receipt button is located on the module a few centimeters away from the indication lamp, it is rather impossible to use the button on the wrong module.

#### 2.5.3. Pick-by-Point

**Figure 6:**
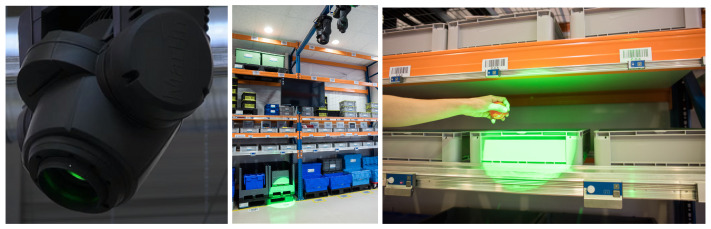
Pick-by-Point location indicator lamp.

The main component of the Pick-by-Point system is a lamp (Figure 6) suspended from the top of the magazine in the middle. The task of the lamp is to indicate with a beam of light the location on the shelf from which the participant should pick up the item. As with the previous two systems, the lamp also shows the exact locations in the same order according to the documents, an example of which is Figure 4.

**Figure 7:**
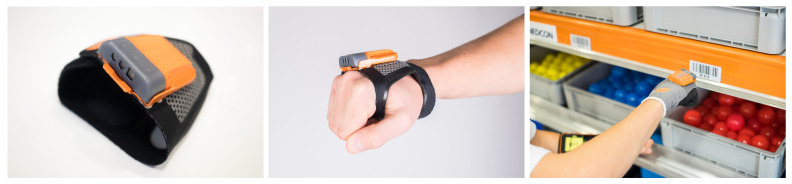
Wrist scanner for Pick-by-Point receipt.

The second component of this system used for receipts is a wrist scanner (Figure 7). Each location has a unique barcode. The barcodes in zones A and B are next to the Pick-by-Light module (Figure 5 and Figure 6 right), so above the bin. In zones C and D, for the lowest-located large containers, the barcodes are on the floor (Figure 2). For bins on shelves, the barcodes are next to the Pick-by-Light modules as in zones A and B, and so above the bin.

The premise is that the participant should first pick up the item, after which they can only scan the barcode. If the wrong code is scanned, the lamp changes its light color from green to red. Once the scanner reads the correct code, the lamp illuminates the following location.

## 3. Results

A total of 71 participants (43 full-time and 28 part-time students) participated in the Pick-by-Light and Pick-by-Point system studies. In contrast, 28 participants (part-time students) participated in the Pick-by-Paper system study. Participants were numbered to identify themselves and enable the measurements’ results to be analyzed in relation to individual participant performance. Due to the identification of the participants, it was possible to carry out tests of the Pick-by-Light and Pick-by-Point systems for paired data. Also, these types of tests could have been carried out to compare times in individual passes.

Shapiro-Wilk tests are performed for each pass of each system. As a result of the tests, with α=0.05 only for pass 4 in Pick-by-Light and pass 5 in Pick-by-Point, there are no grounds to reject the hypothesis $H_{0}$ that the sample belongs to a population with a normal distribution. In the remaining cases, the value of *p*-value is significantly smaller than α. As a result, comparative tests ar performed using the Wilcoxon or Mann–Whitney U test. Accepting the hypothesis $H_{0}$ means that the differences between the compared samples are not statistically significant. In this case, we can state that the order-picking times obtained in the two passes are not statistically significantly different.

### 3.1. Analysis of Order Picking Times

The analysis of order-picking times is carried out using box plots, where participants’ times are marked with black points. The red point distinguishes the order-picking time of the person with experience. On the other hand, the value of this time is given in an oval with a red border. Numerical values with a black border indicate the characteristic points of the box plot, i.e., median, $Q_{1}$ and $Q_{3}$ quartiles, and whiskers. In addition, statistical tests are performed.

Time–pass → *Pick-by-Paper*

**Figure 8:**
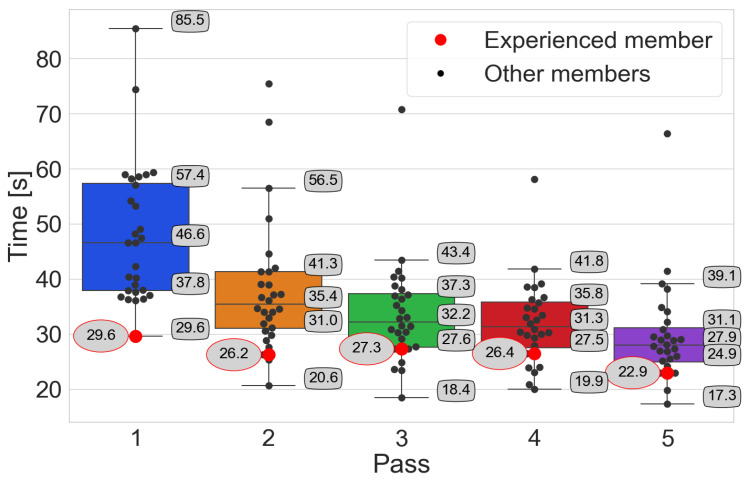
The participants’ achieved order-picking times with the Pick-by-Paper system, emphasizing the person with experience.

In the results of the tests carried out for the Pick-by-Paper system (Figure 8), it can be seen with each successive pass that the median picking time of the participants decreases and that the range of times achieved narrows.

The Wilcoxon test shows a statistically insignificant difference between passes 3 and 4. The hypothesis $H_{0}$ is rejected in the remaining cases with α=0.05. The person with experience achieves some of the shortest times in each pass.

Time–pass → *Pick-by-Light*

**Figure 9:**
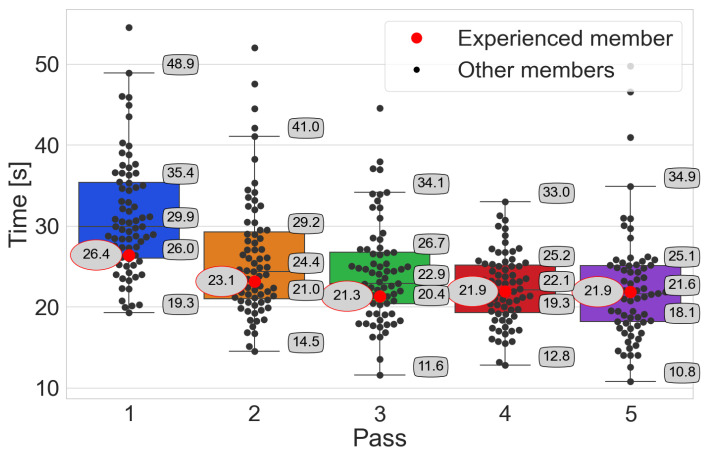
The participants’ achieved order-picking times with the Pick-by-Light system, emphasizing the person with experience.

The results for the Pick-by-Light system (Figure 9) show significantly shorter times than the Pick-by-Paper system. In the following passes, there is a reduction in the median time obtained by the participants. The Wilcoxon one-sided and the two-sided tests for the following passes show that the difference is statistically significant. In the case of the person with the experience, rapid adaptation to the system and stable performance in the following passes can be observed.

Time–pass → *Pick-by-Point*

**Figure 10:**
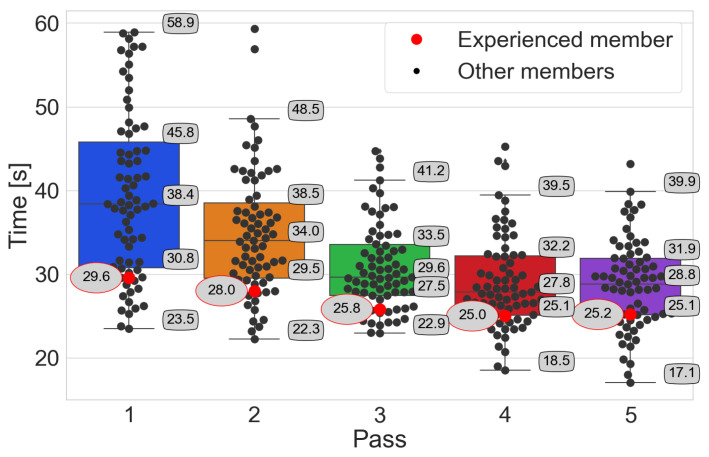
The participants’ achieved order-picking times with the Pick-by-Point system, emphasizing the person with experience.

The Wilcoxon test conducted for consecutive passes of both one-sided and two-sided does not give grounds to reject the hypothesis $H_{0}$ for passes 4 and 5. This may suggest that participants only need four passes to adapt to this system (Figure 10). A person with experience can be inferred to have adopted after only three passes.

All systems combined

**Figure 11:**
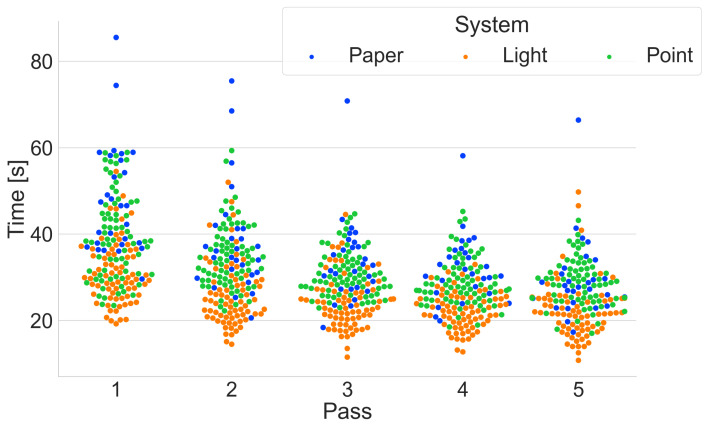
Summary of all times in the following passes, distinguishing between the different systems.

A summary of all the times obtained by the participants (Figure 11) shows a concentration of shorter times for the Pick-by-Light system compared to the other two systems. Many outliers can be seen in the Pick-by-Paper system. This only confirms that the participants have problems finding the location read from the paper document.

Summary of systems by pass

**Figure 12:**
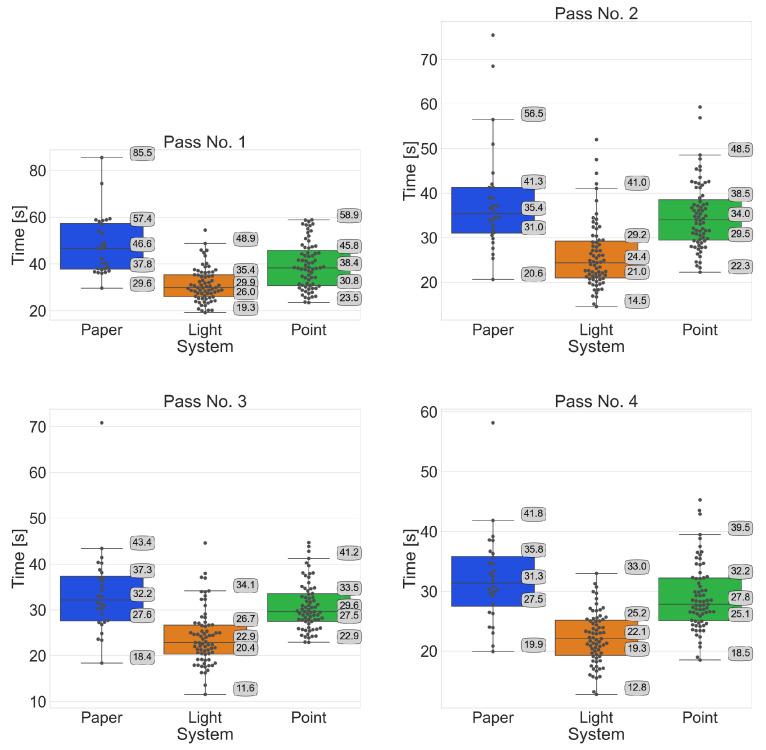

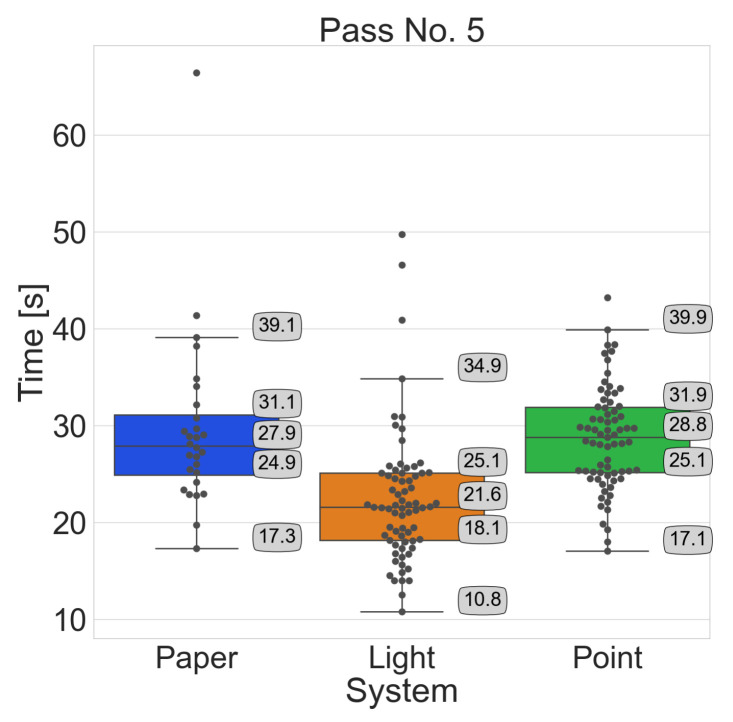
Comparison of the tested systems by pass.

A comparison of the tested systems by pass (Figure 12) confirms that participants with the Pick-by-Light system obtain shorter order-picking times than the other two systems. The Mann–Whitney U test does not warrant rejection of the $H_{0}$ hypothesis for passages 2 and 5 when comparing the Pick-by-Paper and Pick-by-Point systems. However, in the other passes, the Mann–Whitney U test results reveal a statistically significant difference, providing strong evidence for the efficiency of the Pick-by-Light system.

### 3.2. Analysis of Time Differences Between Passes

Time differences between passes

**Figure 13:**
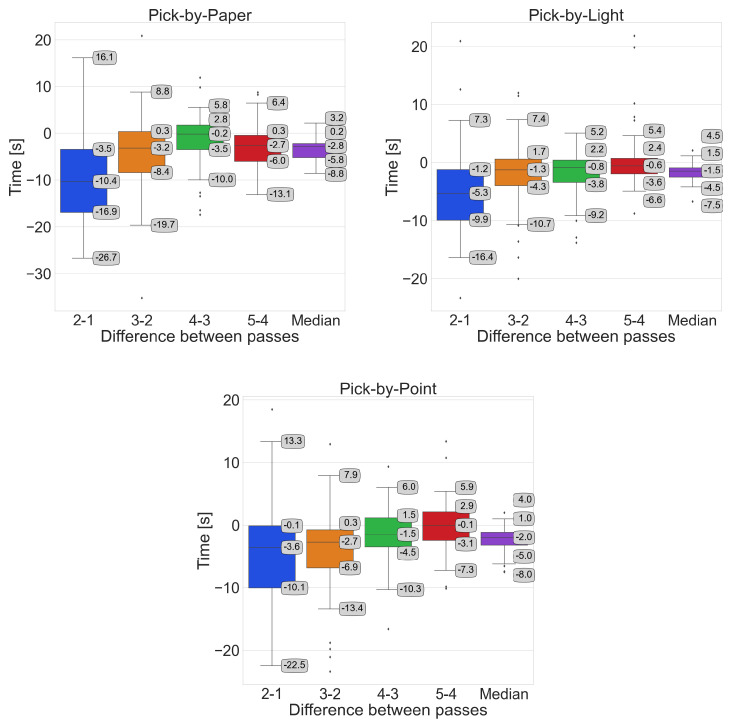
Comparison of the time differences between passes by system.

This indicator is calculated for each participant individually so that the time obtained in pass 2 is subtracted from the time in pass 1, that in pass 3 from pass 2, and so on. The last box (Figure 13) is a picture of the median of the four differences for each participant individually. This indicator is intended to represent the overall trend of participant adaptation in order picking with the help of the individual systems. A negative value, in the context of our study, indicates reductions in times in successive passes.

**Figure 14:**
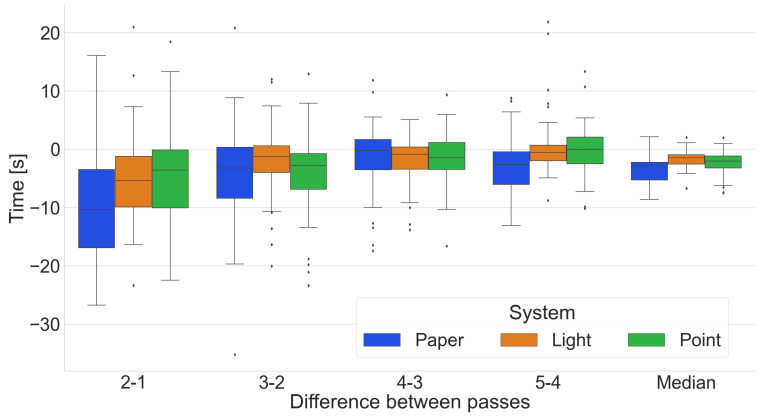
Comparison of time differences between successive passes in the overall summary.

In both Figure 13 and Figure 14, it can be seen by narrowing the range of times obtained that the participants as a group are adopting the systems used. Outliers occurring in several cases signify the cart tipping over on the turn while heading from zone B to zone C and needing to be lifted, or difficulty finding the location in the upper left corner of zone C.

### 3.3. Analysis of Incorrect Pick-Ups

Incorrect pick-ups

During the test, we record instances of incorrect pick-ups. The Pick-by-Paper system has 48 errors, the Pick-by-Light system has 41 errors, and the Pick-by-Point system has 21 errors (Table 1). It is worth noting that the majority of errors in the Pick-by-Light system are due to a common incorrect assumption among participants. They assume that the item bin is above the indicating module and not below it. These errors are mainly made in zones A and B.

Errors are converted to their percentage occurrence regarding all pick-ups within the study of a given system and are summarized in Table 2. For the Pick-by-Paper system, 28 people participate by completing 5 pick-ups in each of 5 orders (1). For the other systems, it is 71 people (2).

(1) $$\frac{48}{28\cdot 5\cdot 5}\cdot 100\%=\frac{48}{700}\cdot 100\%=6.86\%$$

(2) $$\frac{41}{71\cdot 5\cdot 5}\cdot 100\%=\frac{41}{1775}\cdot 100\%=2.31\%$$

Another indicator is the average number of errors per participant (*P*—participant, Ps—participants) as presented in Table 3. The calculations are performed according to (3) and (4). This indicator shows that the Pick-by-Paper system averages 1.71 errors per participant, a high value.

(3) $$\frac{48\;\mathrm{errors}}{28\;\mathrm{Ps}}=1.71$$

(4) $$\frac{41\;\mathrm{errors}}{71\;\mathrm{Ps}}=0.58$$

Summarizing the total number of errors during the test, the Pick-by-Point system proves the most resilient. This system does not allow passing to the following location after scanning the wrong barcode, but in such a case, the error is recorded.

Time by number of errors made–*Pick-by-Paper*

**Figure 15:**
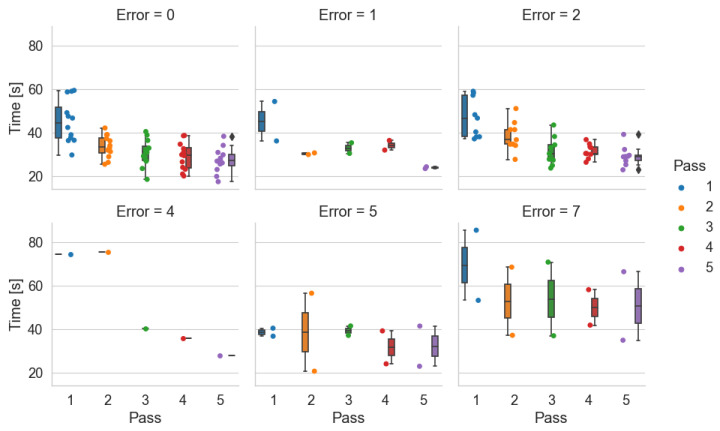
Order-picking time per pass broken down by number of errors made using the Pick-by-Paper system.

**Figure 16:**
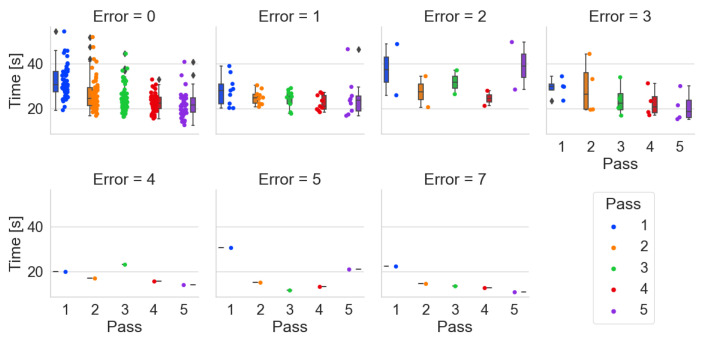
Order-picking time per pass broken down by number of errors made using the Pick-by-Light system.

**Figure 17:**
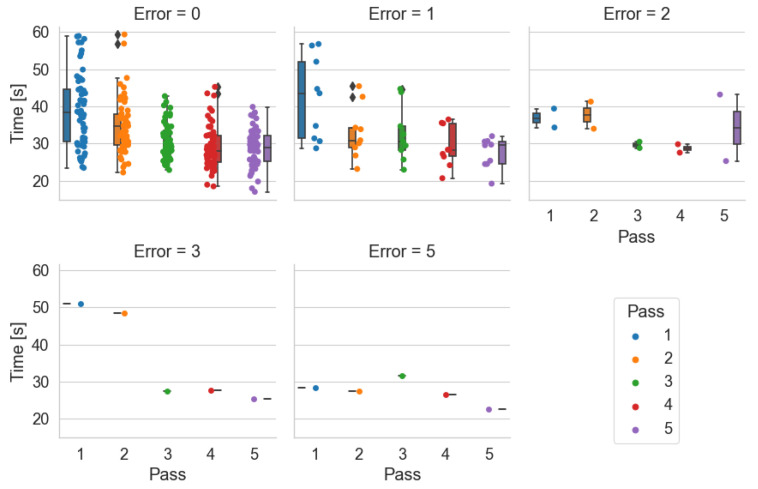
Order-picking time per pass broken down by number of errors made using the Pick-by-Point system.

During the study, the sum of errors made by a given participant in all passes within a given system is counted. Figure 15, Figure 16 and Figure 17 show the participant times obtained in each pass with a breakdown of the number of errors made. The graph Error=4 in Figure 15 shows the times obtained by one participant in each pass with four errors. This graph indicates that the participant lost much time during the first and second orders. In the others, he accelerated. However, deducing from the information collected the passes in which he made mistakes is impossible.

The Pick-by-Paper system is the only tested system that does not have an element of receipt of the pick-up of the item and possible verification of correctness as in the case of Pick-by-Point. With the help of this system, two people made 7 errors, and two made 5 errors. During the test, participants with this system were unaware they were making errors. They were not informed of this by the person counting the errors. From Figure 15, it can be deduced, as indicated by the times obtained, that the people who made 7 errors were not in a hurry. They had difficulty finding the location indicated on the document (Figure 4).

Time by number of errors made–*Pick-by-Light*

In the case of the Pick-by-Point system, those making the most errors of 4, 5, and 7 were in a hurry to complete the order (Figure 16). These participants prioritized a short order-picking time.

Time by number of errors made–*Pick-by-Point*

During order picking with the Pick-by-Point system, one person made 3 and one made 5 errors (Figure 17). Two people made two errors each. In this method, the participants’ problem was adapting to the wrist scanner and placing barcodes on the shelves.

### 3.4. Survey Results

Participant evaluations for the tested systems

The survey’s primary objective was to gather participants’ perspectives on the indicating and receipt systems, emphasizing the pivotal role of employee comfort in workplace efficiency. The indication’s clarity and the receipt system’s convenience significantly influence an individual’s work. Employees working in comfortable conditions are more likely to complete tasks quickly and with fewer errors, highlighting the crucial role of their comfort in the workplace.

The need for unambiguous indication systems cannot be overstated. It is not good for the employee to look for a shining light or an illuminated location. Equally important is to assess whether the system causes rapid employee fatigue or is genuinely comfortable and conducive to efficient work.

The same is true of the receipt system. The main objective was to obtain a subjective evaluation based on the participants’ feelings. In this case, they also wanted to know which system was more comfortable, emphasizing the necessity of their involvement in the evaluation process.

After picking the 5 orders, participants gave their impressions in an ongoing survey. From the responses received (Table 4), 27 participants out of 53 for Pick-by-Light and 24 participants out of 51 for Pick-by-Point could quickly see the location indicated by the system, which is a promising start. Moreover, for 31 people, both systems were easy to follow, indicating a high potential for usability. In total, 13 and 12 responded that the systems were convenient. However, it is important to note that there were also people for whom the systems were tiring and cumbersome.

Comments submitted to the system *Pick-by-Point*

The participants also expressed their perceptions in a loose statement on two questions (Table 5). A total of 29 people reported problems with the scanning. Despite being able to type in any statement, the participants’ comments were very similar. The problem was that the scanner had to be placed right from the barcode. If the scanner was too close or far away, the receipt failed, and the system did not indicate the new location. The scanner receipt problem also had an impact on order-picking times.

Comparative survey–*indicating systems*

As with the previous survey, participants were not obliged to respond but encouraged to do so; hence, not all were completed. Participants were very divided in the comparison survey (Table 6). A total of 29 out of 62 participants found the Pick-by-Light indicating method the most intuitive and fastest to learn. On the other hand, 32 participants identified the Pick-by-Point system as the most intuitive and 31 identified it as the quickest to learn. Interestingly, some participants found both systems to be tiring.

Comparative survey–*receipt systems*

For the survey on receipt systems, 39 out of 62 participants (62.9%) indicated the convenience of the proglove system, i.e., using a wrist scanner (Table 7). The others favored the button system on the Pick-by-Light module.

## 4. Conclusions

Three systems were examined to support the manual picking process, i.e., Pick-by-Paper, Pick-by-Light, and Pick-by-Point. In addition to the first system, the other two were supplemented by a receipt system. In the case of the first, it was based on a button on the module, while the second used a wrist-based barcode scanner. Each system had its strengths and weaknesses, directly impacting the indicators studied. The stated aims of the study were to examine the order-picking time, the speed of participant adaptation to the systems, the number of errors made, and the participants’ opinions of the systems.

**Figure 18:**
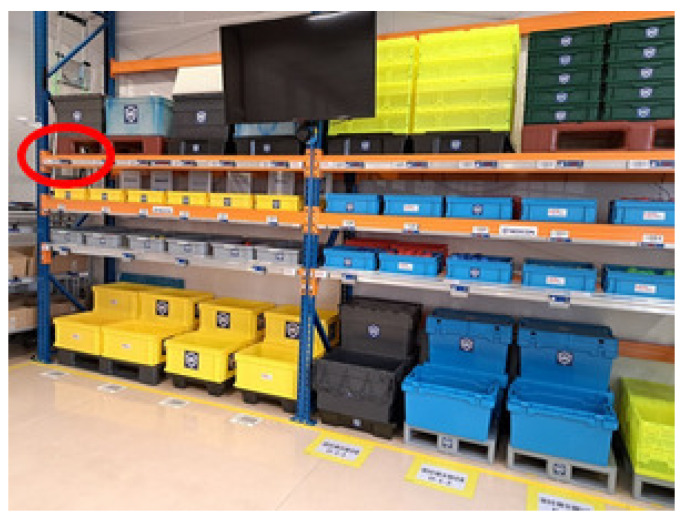
A location difficult for participants to see.

- Pick-by-Light proved to be the fastest order-picking and employee adaptation (learning) system according to the measurements, while Pick-by-Point was the most resistant to errors made.
- The impact on the number of incorrect pick-ups in the Pick-by-Light system was the difficulty for participants to remember that the container is under the indicating module and not above it. This was particularly the case in zones A and B, i.e., the initial order-picking phase. In this system configuration, the receipt is indicated via a button on the module. The system for indicating an incorrect pick-up of an item, i.e., flashing a light, is not practical. The system would require item identification, e.g., using a barcode directly on the item, an infrared curtain, or an RFID system with a tag on each item.
- The time of participant adaptation to the wrist scanner influenced the order-picking time with the Pick-by-Point system. Many participants expressed this in the survey, indicating difficulties with the scanner reading the barcode.
- The person with the experience of 9 years of working in the picking process did not make any mistakes. In addition, the person quickly adapted to the respective system after only three orders. In her professional work, this person worked mainly with the Pick-by-Voice system.
- Participants found it difficult to locate the upper left corner of the high rack in zone C (Figure 18). In particular, during the Pick-by-Light implementation, they did not notice the light being on.
- In future studies, it would be helpful to count the number of participants’ incorrect pick-ups for each pass rather than as the sum of all passes within a given system. Such an approach could show the relationship between the participant order-picking speed and the number of errors made within a given system.
- The results of the surveys indicate the importance of introducing more specific training to sensitize new employees to prevalent mistakes and the locations of common mistakes.

Future work

- In this study, one ball was always taken. In future studies, the system could be extended to include a system for the number of balls taken from a given location. Such a system could be Pick-by-Watch. This system uses a smartwatch or a mobile device the size of a smartphone placed on the hand. Another system to support Pick-by-Point could be a tablet mounted on a cart.
- The study was conducted in a laboratory setting. One participant was working at any given time. The laboratory has two lamps, allowing two people to work simultaneously.
- The laboratory also has zone lighting. Each of the four zones can be individually illuminated or extinguished. A server controls the zone lighting. The same server controls the Pick-by-Point lamps and the Pick-by-Light modules. The zone lighting can support these systems. In addition, the zonal lighting can be dimmed steplessly, allowing the possibility to conduct tests at different light levels.
- In addition, the execution of multiple orders by a single employee can be tested. A cart equipped with 12 bins can be used for this purpose. In addition, the cart is equipped with Pick-by-Light modules for each bin. The modules can also indicate the quantity of products to be picked up for a given order. The indication of the items on the shelf can be performed with the Pick-by-Point or Pick-by-Light system. The receipt can be indicated with a scanner or a button on the module.

## Figures and Tables

**Table 1:** Number of total incorrect pick-ups by system.

System	No. of Participants	No. of Errors
Pick-by-Paper	28	48
Pick-by-Light	71	41
Pick-by-Point	71	21

**Table 2:** Percentage of incorrect pick-ups in relation to all pick-ups.

System	Errors
Pick-by-Paper	6.86%
Pick-by-Light	2.31%
Pick-by-Point	1.18%

**Table 3:** Number of incorrect pick-ups per participant.

System	Errors/P
Pick-by-Paper	1.71
Pick-by-Light	0.58
Pick-by-Point	0.30

**Table 4:** Survey results for systems Pick-by-Light and Pick-by-Point.

1. Locations indicated by the system	**Light**	**Point**
(a) I had to look, think for a while	10	8
(b) I was able to see right away	27	24
(c) it got easier with every step	16	16
(d) a difficult start	0	3
2. The tested system appears to be in the long term		
(a) cumbersome	3	5
(b) tiring	6	2
(c) easy to learn	31	31
(d) comfortable, compatible with my aptitude	13	12

**Table 5:** Loose answers to two questions.

1. Reasons for discomfort in the system:	
accuracy of receipts (proglove)	10
inadequate fit of the scanner in the hand	3
right-hand scanner	2
2. In my opinion, the disadvantage of the tested system is:	
accuracy of receipts (proglove)	17
receipt method	2
fatigue of one hand	1

**Table 6:** The results of the comparative survey of indicating systems.

1. In my opinion the most intuitive system of indicating is:	
Pick-by-Light	48.6% (29 of 62)
Pick-by-Point	51.4% (32 of 62)
Pick-by-Paper	(1 of 25)
2. In my opinion the most tiresome system of indicating seems to be:	
Pick-by-Light	36.7% (22 of 60)
Pick-by-Point	35.0% (21 of 60)
Pick-by-Paper	(17 of 25)
3. In my opinion the quickest learned system of indicating seems to be:	
Pick-by-Light	46.8% (29 of 62)
Pick-by-Point	50.0% (31 of 62)
Pick-by-Paper	(2 of 25)

**Table 7:** The results of the comparative survey of receipt systems.

1. In my opinion the most comfortable receipt system is:	
button on the module	37.1% (23 of 62)
Proglove	62.9% (39 of 62)
2. In my opinion the most cumbersome receipt system seems to be:	
button on the module	62.9% (39 of 62)
Proglove	37.1% (23 of 62)
3. In my opinion the quickest learned receipt system seems to be:	
button on the module	38.7% (24 of 62)
Proglove	61.3% (38 of 62)

## Data Availability

https://github.com/ResearchOfMan/Paper-Light-Point.
